# A Survey on Data-Driven Approaches for Reliability, Robustness, and Energy Efficiency in Wireless Body Area Networks

**DOI:** 10.3390/s24206531

**Published:** 2024-10-10

**Authors:** Pulak Majumdar, Satyaki Roy, Sudipta Sikdar, Preetam Ghosh, Nirnay Ghosh

**Affiliations:** 1 Department of Computer Science and Technology, Indian Institute of Engineering Science and Technology, Shibpur, Howrah 711103, India; 2022csm007.pulak@students.iiests.ac.in; 2 Department of Mathematics, University of Alabama, Huntsville, AL 35899, USA; sr0215@uah.edu; 3 Department of Computer Science and Technology & Computer Science and Information Technology, University of Engineering & Management, Kolkata 700160, India; sudipta.sikdar@uem.edu.in; 4 Department of Computer Science, Virginia Commonwealth University, Richmond, VA 23284, USA; pghosh@vcu.edu

**Keywords:** wireless body area networks, wearable biosensors, photoplethysmography, redundancy, energy efficiency, robustness, machine learning

## Abstract

Wireless Body Area Networks (WBANs) are pivotal in health care and wearable technologies, enabling seamless communication between miniature sensors and devices on or within the human body. These biosensors capture critical physiological parameters, ranging from body temperature and blood oxygen levels to real-time electrocardiogram readings. However, WBANs face significant challenges during and after deployment, including energy conservation, security, reliability, and failure vulnerability. Sensor nodes, which are often battery-operated, expend considerable energy during sensing and transmission due to inherent spatiotemporal patterns in biomedical data streams. This paper provides a comprehensive survey of data-driven approaches that address these challenges, focusing on device placement and routing, sampling rate calibration, and the application of machine learning (ML) and statistical learning techniques to enhance network performance. Additionally, we validate three existing models (statistical, ML, and coding-based models) using two real datasets, namely the MIMIC clinical database and biomarkers collected from six subjects with a prototype biosensing device developed by our team. Our findings offer insights into strategies for optimizing energy efficiency while ensuring security and reliability in WBANs. We conclude by outlining future directions to leverage approaches to meet the evolving demands of healthcare applications.

## 1. Introduction

Wireless Body Area Networks (WBANs) represent wearable healthcare technology, facilitating seamless communication among miniature sensors and devices strategically positioned on or implanted within the human body. These networks hold immense importance, as they enable real-time monitoring of physiological parameters, providing valuable insights into an individual’s health status [1]. WBANs play a pivotal role in various medical applications, ranging from continuous health monitoring and early disease detection to the facilitation of personalized healthcare solutions. Through the wireless connections of biomedical sensors, actuators, and wearable devices, WBANs empower individuals and healthcare professionals with instantaneous access to critical patient data, paving the way for targeted diagnosis, timely interventions, and enhanced patient care [2]. Beyond healthcare, the versatility of WBANs extends to diverse domains such as sports monitoring, wellness tracking, and human–computer interaction, underscoring their significance in shaping the future of connected and personalized healthcare monitoring ecosystems [3,4,5,6].

WBANs have many applications, from hospital patient monitoring to enhancing military performance. *First*, the healthcare industry offers one of the most promising applications of WBANs [7]. Biosensors deployed in the human body can monitor physiological parameters, allowing physicians, family members, and diagnostic centers to access these signals remotely. Real-time observation of body signals can detect critical events such as heart attacks and strokes. WBANs can improve living conditions through the use of devices like hearing aids and implants. *Second*, athletes and sports personnel can benefit from WBANs by monitoring vital body parameters such as *SpO_2_*, heartbeat, blood pressure, and glucose levels. WBANs can enhance realism in the entertainment sector by tracking posture, facial expressions, and movements, offering a more immersive experience [8]. *Third*, *WBAN* technology is also vital in military operations, contributing to network-enabled capability efforts to enhance military effects through the use of information systems [9]. Sensors monitor vital signs and environmental conditions, helping soldiers avoid threats, while inter-WBAN communications and spatial localization protect sensitive information.

WBAN communication suffers from several challenges [10]. The primary aim of supporting life-saving medical applications within wireless body area networks (WBANs) underscores the critical importance of *Quality-of-Service* (QoS) standards, as measured in terms of data loss and communication latency in a delay-sensitive environment [11]. The challenge lies in configuring QoS to suit application needs while ensuring fair bandwidth sharing among co-located WBANs and enabling graceful service degradation. Limited device memory further complicates matters, necessitating effective error detection and correction schemes. *Reliability* and *robustness*, as determined in terms of the ability of the network to meet the QoS standards despite failures, are particularly crucial for ensuring the success of these networks in critical medical applications. The potential for lost or corrupt alarm/alert packets due to unreliable wireless networks encompasses the need for efficient acknowledgment and retransmission mechanisms. Integrating *heterogeneous wireless networking technologies* poses a significant challenge in realizing the full potential of WBANs. Integrating low-cost, limited-range, high-capacity wireless local area network (WLAN) and personal area network (WPAN) infrastructure for indoor connectivity, alongside lower-capacity, longer-range cellular infrastructure for outdoor connectivity, requires issues of seamless roaming and end-to-end QoS to be addressed. The gateway device, acting as a bridge between WBANs and infrastructure networks, must facilitate smooth data transfer, storage, and offloading during non-real-time applications. *Security* concerns in WBANs extend beyond conventional considerations due to limited resources, lack of user interface, unskilled users, and global roaming. Traditional security and privacy mechanisms are often unsuitable for WBANs, necessitating the development of resource-efficient methods.

In WBANs, addressing the inherent spatial and temporal patterns in biomedical data streams is crucial for enhancing the reliability and robustness of data transmission [12]. By carefully analyzing these correlations and the consequent redundancy, systems can better mitigate the impact of potential disruptions, leading to improved performance. Specifically, excessive redundancy can introduce energy overheads and increase the vulnerability surface, compromising security, while insufficient redundancy may jeopardize *quality of service* (QoS) during component failures [13]. Therefore, it is essential to control the spatiotemporal data correlation to enhance reliability and robustness without degrading QoS, compromising biomedical information security, or reducing energy efficiency, demanding a nuanced approach that considers the specific requirements of the wireless communication system for clinical applications, ensuring resilience to disruptions.

Spatiotemporal data correlation influences WBAN QoS in the following specific ways. *Firstly*, it is crucial in addressing the challenges faced by WBANs, playing a significant role in maintaining quality of service (QoS). By offering alternative routes for data transmission, redundancy helps mitigate delays and losses, ensuring consistent performance. However, excessive redundancy can reduce network lifetime and potentially lead to adverse clinical outcomes due to decreased energy efficiency, which is paramount in WBANs. *Secondly*, it enhances the reliability of communication. Backup paths and retransmission mechanisms ensure the delivery of critical information, even in the presence of interference or packet loss, thereby contributing to network robustness. *Thirdly*, in scenarios involving heterogeneous wireless networking technologies, redundancy provides added reliability. Data replication across multiple paths ensures seamless network functionality, even when individual links fail. *Lastly*, redundant security measures, including multiple layers of authentication and encryption, enhance data protection. Lastly, redundant data storage and transmission mechanisms safeguard against potential security breaches or data corruption.

**Figure 1:**
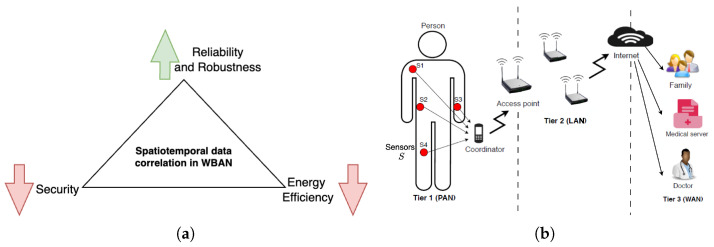
Spatiotemporal data correlation in wireless body area networks (WBANs). (**a**) Effect of redundancy on security, reliability, robustness, and energy efficiency. (**b**) System model showing the communication among sensors, coordinator nodes, and access points.

We provide a comprehensive survey, encompassing an in-depth review of data-driven approaches aimed at enhancing energy efficiency, security, reliability, and robustness to failures (see Figure 1a). We explore various device placement and routing approaches that leverage these patterns to optimize network performance. Furthermore, we delve into existing strategies for calibrating sampling rates, as well as the application of machine learning (ML) and statistical learning techniques in WBANs, highlighting their contributions to the efficient, secure, and reliable operation of these time-critical WBAN applications.

We implement a validation framework to conduct a detailed analysis of the pros and cons of representative approaches within three distinct classes, namely ML, statistical methods, and coding-based approaches. Our evaluation, based on real biomedical datasets including the MIMIC dataset [14], as well as health markers such as heart rate and blood oxygen levels collected from 6 subjects using the prototype device designed by our team, provides insights into how each approach addresses data-related challenges in WBANs. This comparative analysis offers a perspective on their practical applicability and performance. We conclude with an outline of future directions for leveraging the spatiotemporal data correlation to optimize WBAN communication. This includes potential advancements in integrating redundancy with emerging technologies such as emerging ML models and adaptive algorithms to further enhance the efficiency, security, and reliability of WBANs.

## 2. Communication Architecture of WBANs

The communication architecture of wireless body area networks (WBANs) can be categorized into three tiers, namely intra-WBAN communication, inter-WBAN communication, and beyond-WBAN communication [15]. Each tier contributes to efficient data transmission and system reliability. Figure 1b demonstrates these communication tiers within a component-based WBAN system. In this figure, devices are dispersed across the body in a centralized network architecture, with the exact location of each device tailored to specific applications. Given that the body is often in motion (e.g., running or walking), the ideal placement of sensor nodes can vary, making WBANs inherently dynamic.

### 2.1. Types of Nodes

WBAN nodes are classified into three types based on communication characteristics [16,17].

- *Coordinator or personal devices* serve as a central hub for collecting signals from sensors and forwarding information to recipients such as physicians, cloud services, or external devices. For experimental validation (discussed in Section 4), we treat the coordination node as the *base station (BS)*.
- *Sensors* are the primary components responsible for acquiring physiological parameters from the body. They can measure various health parameters, environmental conditions, and biokinetics. Sensors are deployed in diverse locations, such as wristwatches, smartphones, and various body parts.
- *Actuators* perform specific actions based on data received from sensor nodes, returning feedback by executing tasks such as administering medicine or adjusting glucose levels in response to sensor data.

IEEE 802.15.6 [18,19] presents another categorization based on sensor node placement on or within the human body, distinguishing between *implant*, *body-surface*, and *external* nodes. IEEE 802.15.6 defines key specifications for wireless body area networks (WBANs) to ensure efficient and reliable communication in medical and non-medical applications. The bit rate must range between 10 kbps and 10 Mbps, supporting a maximum of 256 nodes per network. The packet error rate (PER) should remain under 10% in approximately 95% of the best-performing links, while node join and leave operations must occur in less than 3 s. To maintain reliable communication, even with mobile nodes, the maximum tolerable latency is 125 ms for medical applications and 250 ms for non-medical applications, with jitter limited to 50 ms. Additionally, WBANs should comply with a Specific Absorption Rate (SAR) of 1.6 W/kg in 1 g of body tissue, with devices transmitting at 0.1 mW and a maximum transmission power of 1.0 mW. WBANs must also support interoperability between devices following different standards and be capable of power-saving operations while ensuring priority services and self-healing capabilities for quality of service (QoS).

### 2.2. Hierarchical Communication System

The communication architecture of WBANs is a multi-tiered system designed to ensure seamless and efficient data transmission across different levels of operation. Each tier, from intra-WBAN communication to beyond-WBAN communication, plays a critical role in enhancing the functionality, reliability, and scalability of WBANs in various applications, particularly in health care and medical monitoring systems.

#### 2.2.1. Tier 1: Intra-WBAN Communication

Tier 1 focuses on communication within a single WBAN, covering interactions among sensor nodes located in and around the human body within a transmission range of approximately 2 m. The sensors capture physiological signals and transmit them to an *access point* (AP) in tier 2.

#### 2.2.2. Tier 2: Inter-WBAN Communication

Tier 2 deals with communication between the BS and multiple access points (APs). These APs can be part of the infrastructure or strategically positioned in dynamic environments to manage emergencies. The primary goal is to interconnect WBANs with other networks, such as cellular networks and the Internet, facilitating easy access in everyday life. This tier’s communication is divided into the following two subcategories:

- **Infrastructure-based Architecture**: This common architecture is used in many WBAN applications. It supports dynamic deployment within confined spaces like hospitals and offers centralized management and security control, functioning as an application-specific database server.
- **Ad hoc Architecture**: In this architecture, multiple APs transmit information within medical centers, forming a mesh network. This configuration allows for flexible and rapid deployment and extended radio coverage through multi-hop dissemination and supports patient mobility. The coverage range extends up to 100 m, making it suitable for both short-term and long-term setups.

#### 2.2.3. Tier 3: Beyond-WBAN Communication

Tier 3 encompasses communication beyond the WBAN, particularly in metropolitan areas. A gateway device, such as a personal digital assistant (PDA), bridges the connection between tiers 2 and 3, linking the Internet to a Medical Server (MS) in specific applications. The design of this tier is highly application-specific. In medical environments, a database forms a critical component of tier 3, storing clinical profiles and enabling notifications of emergency statuses to doctors or patients via the Internet or SMS. Tier 3 also facilitates the retrieval of essential patient information for treatment purposes. Depending on the application, the BS in tier 1 may utilize GPRS/3G/4G technology instead of directly communicating with an AP.

## 3. Methodologies

**Figure 2:**
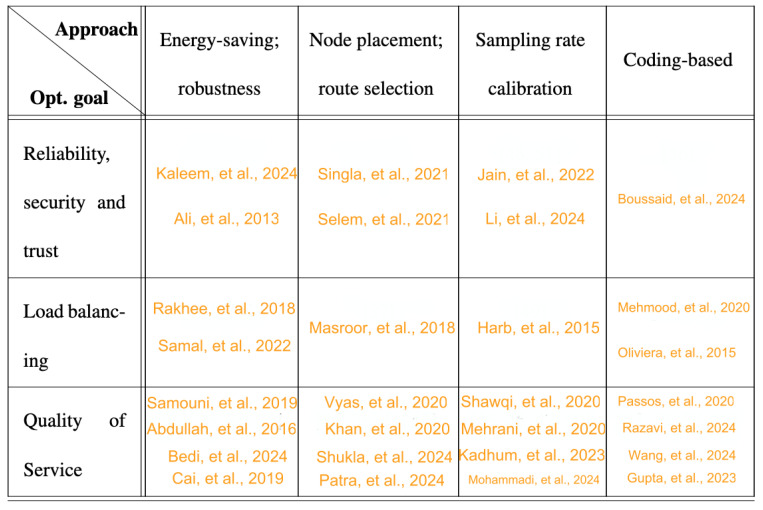
Summary of methodologies surveyed based on different optimization goals and approaches.

The surveyed methodologies (summarized in Figure 2) utilize approaches such as (1) energy saving and robustness maximization, (2) intelligent node placement and route selection, (3) sampling-rate calibration, and (4) coding-based techniques to achieve the following optimization goals.

- *Reliability, Security, and Trust.* Security is paramount in WBANs, where transmitting sensitive health data demands protection against unauthorized access and attacks. Data-driven approaches have been employed to ensure the integrity and confidentiality of health information by detecting and mitigating security breaches in real-time. This category encompasses studies that explore how such techniques can mitigate security threats, detect and recover from attacks, and establish trust in WBAN communication. Examples include secure key management, authentication protocols, and intrusion detection systems driven by data insights.
- *Load Balancing.* Load balancing is critical in WBANs, where multiple sensors collaborate to monitor physiological parameters, optimizing the distribution of computational and communication load across the network, ensuring resource efficiency, and preventing overload on specific components. The studies in this category investigate how data-driven mechanisms contribute to effective load balancing in WBANs. Topics include dynamic resource allocation, task offloading, and adaptive strategies to maintain an even distribution of network resources based on real-time data.
- *QoS (Energy Efficiency and Robustness).* Quality of service (QoS) is essential for the success of WBANs, particularly in healthcare applications requiring real-time monitoring. State-of-the-art approaches significantly enhance QoS by improving the robustness of data transmission and ensuring overall network reliability. Reliability, in this context, refers to the network’s ability to withstand and overcome faults, including energy depletion of nodes and network failures arising from human mobility. These approaches safeguard against potential disruptions and facilitate patient mobility, particularly when patients move beyond Wi-Fi network coverage [20].

### 3.1. Energy Efficiency and Robustness to Failures

Efficient energy utilization is a critical aspect of a heterogeneous wireless communication system, directly influencing its reliability and longevity. State-of-the-art approaches focus on maximizing system lifetime by designing ultra-low-power radio transceivers, enabling devices to sleep for extended periods and thereby reducing the duty cycle. Additionally, choosing low-power WBAN technology further contributes to energy conservation [21,22,23,24,25]. Addressing failures caused by energy depletion, technical faults, or attacks is equally vital to maintain system robustness [26,27,28]. Kaleem et al. proposed a method that uses Support Vector Machine (SVM) classifiers and a neuro-fuzzy inference system (ANFIS) to detect interference and malicious sensor nodes in WBANs. By identifying and correcting sensor failures, the system prevents energy wastage due to repeated transmission errors, enhancing overall efficiency [29]. Similarly, Bedi et al. introduced the Thermal-aware, Energy-efficient, Congestion-aware Routing Protocol (TECRP) to balance energy use, reduce node temperature, and avoid congestion in both inter- and intra-WBAN communications. Their multi-objective approach to optimizing energy consumption and managing node temperature ensures minimal energy depletion, extending network lifetime [30].

**Figure 3:**
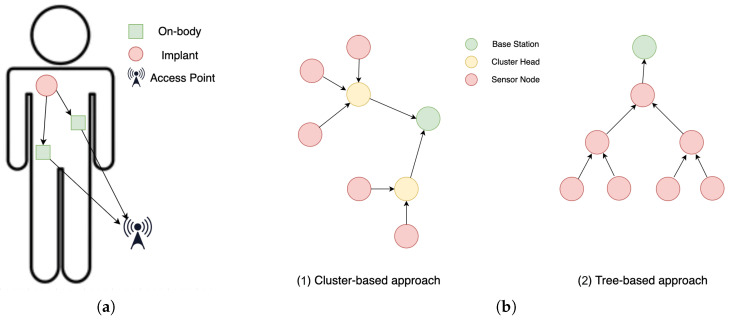
Energy efficiency and strategic node placement in WBANs. (**a**) Communication of an implant WBAN with the wireless access point. (**b**) Energy-efficient protocols for biosensor networks. (This figure was redrawn from [35]).

In the domain of WBAN communications, characterized by time-critical data delivery, *implanted devices* have been developed to ensure reliable and energy-efficient medical data transmission, particularly under conditions of path loss and deep fading. Implant WBANs, as shown in Figure 3a, facilitate communication between an implanted device (source) and an off-body access point (destination) through on-body devices (relays). Unlike on-body devices, implanted devices encounter significant path loss in fading environments while requiring high reliability, low power consumption, and extended transmission periods. To address this, joint relay selection and power control strategies are often employed, prioritizing energy efficiency over latency [31]. To further secure and enhance energy efficiency in WBANs, an inexpensive key agreement scheme has been proposed for cluster-based intra-WBAN and inter-WBAN communications involving resource-constrained sensor nodes [32]. Moreover, several studies have focused on optimizing network performance by reducing traffic redundancy and its associated energy consumption. For instance, an ant colony-based energy-efficient algorithm utilizes clustering to manage data packet transmission, balance network load, and extend network lifetime [33]. Another study aimed to improve WBAN throughput by minimizing packet drops and implementing a prioritization scheme based on packet priority and source location [34].

### 3.2. Optimized Placement and Coordinated Routing Approaches

An autoregression-based prediction model was proposed to determine the optimal data transmission rate from sensors placed at various locations on the human body to coordinator nodes. This approach minimizes energy overhead while maintaining acceptable prediction errors [36]. Additionally, the integration of multiple coordinators or gateway nodes in wireless body area networks (WBANs) has been suggested [37]. These nodes are designed to receive data from sensors, aggregate them to enhance efficiency, and transmit them to the base station for centralized processing. The deployment of multiple coordinator nodes effectively extends the network’s operational lifespan and enhances reliability by providing redundancy and ensuring continuous data transmission. Moreover, intelligent node placement has been highlighted to curb data overhead and improve network lifetime by minimizing the distance between communicating nodes [38]. Another approach aimed at minimizing communication overheads within WBANs was introduced in [35]. This strategy comprises the following two routing methods to optimize energy efficiency:

- The *cluster-based approach* involves the strategic selection of cluster heads (CHs) based on the density of biosensors within the network. CHs are identified before node assignment and are responsible for aggregating and transmitting data to the base station (BS). This clustering mechanism streamlines communication and facilitates the efficient organization of biosensors, ensuring an optimal distribution of responsibilities within the network (see Figure 3b(1)).
- In the *tree-based approach*, a hierarchical structure is proposed, where the BS serves as the root of the tree, with the nearest biosensors acting as its children. Through an iterative process, each biosensor is assigned to a designated parent within the tree, creating a structured hierarchy for data transmission. This method leverages the tree structure to systematically push data from biosensors to the BS, promoting an organized and resource-efficient flow of information (see Figure 3b(2)).

Variants of the cluster-based approach have also been proposed. For instance, a clustering-based energy-efficient WBAN focuses on reliable data transmission within a many-to-one stream model, addressing the critical need for resilience in healthcare applications. The proposed mechanism, termed the Network Coding-based Fault-tolerant Mechanism (NCFM), employs a two-step approach [39]. *First*, a greedy grouping algorithm partitions the network topology into smaller logical units. Next, a spanning tree is constructed using random linear network coding, facilitating the creation of linearly independent coding combinations. *Another* paper introduced a method for determining cluster heads in WBANs, emphasizing effective data communication in healthcare monitoring systems. The proposed method considers factors such as residual energy levels and node positions to designate cluster heads, which act as relay nodes for the transmission of messages from surrounding sensor nodes to the base station. By utilizing probability distribution, specific energy, network density, and distance from base stations, this method optimizes the selection of cluster heads. Simulations revealed that this approach increases network lifetime and decreases end-to-end delay compared to the LEACH protocol [40].

Shukla et al. presented an energy-efficient solution for hub node placement in WBANs, using the Whale Optimization Algorithm (WOA) to find the optimal location for the central hub. The WOA minimizes the energy spent by biosensor nodes by reducing the need for multiple trial-and-error placements, speeding up the process compared to traditional methods. The algorithm leverages a population of candidate solutions, or “whale search agents”, that iteratively converge on the best hub location with minimal network energy consumption [41]. Patra et al. proposed a Free Search Krill Herd (FSKH) algorithm for optimization of relay node placement in WBANs, focusing on energy efficiency. They complemented this with a routing protocol based on the Harmony Search (HS) algorithm, further improving the reliability and energy conservation of data transmission [42]. Lastly, other studies have addressed the joint challenge of security and energy-efficient routing. One approach aims to secure data transmission using particle swarm optimization for the selection of the next hop [43]. Similarly, an optimization-based framework aims to improve throughput, prolong node lifetime, and minimize redundancy through synchronized communication and a handover mechanism among the coordinator nodes (CNs). Specifically, two-hop communication was enforced to minimize packet drops under worst-case scenarios where the communicating node is outside the range of the CNs [44].

### 3.3. Sampling Rate Calibration and Machine and Statistical Learning

Calibrating sampling rates is another key aspect of managing energy expenditure in WBANs. A two-step energy-efficient health monitoring approach [45] first optimizes the measured vital signs by eliminating redundancy, then calibrates sampling rates based on risk scores derived from the measurements. Similarly, another model predicts sampling rates based on patient activity and risk, utilizing an adaptive neuro-fuzzy inference system and long short-term memory to forecast future sampling rates and health measurements [46]. A hierarchical approach further reduces redundancy at edge sensors by adapting sampling rates, while edge devices aggregate the information from these sensors. This approach incorporates a support vector machine (SVM) to make informed decisions about patient health [47]. In another two-step health monitoring system [48], the following energy-saving protocol was proposed:

1. For any incoming value (*x*) in a stream of data with mean (*m*) and standard deviation (σ), a Z score is calculated to determine its deviation from the mean, i.e.,
  (1) $$Z=\frac{x-m}{\sigma }$$
  If *Z* is outside reasonable limits $\left[l_{\tau },h_{\tau }\right]$, the data are discarded as faulty. It is considered *interesting* if $\Delta Z=|Z-Z_{previous}|$ is below a prespecified threshold; otherwise, it is marked as *uninteresting*.
2. The isolation forest method is employed to detect any anomalies that could indicate a potentially adverse medical condition requiring immediate clinical attention from the filtered data.

Another statistical approach based on a one-way ANOVA model and Fisher test operates in the following two phases: aggregation and transmission [49]. This method first captures data in time slots and calculates the difference between consecutive values ($|m_{i}-m_{i+1}|$). If this difference exceeds a predefined threshold ($t_{\tau }$), it is retained as a new value; otherwise, the new value is considered redundant, and the frequency of the old value is increased. During the transmission phase, the sensor node evaluates the similarity between data captured over multiple periods using the ANOVA model and Fisher test. Only dissimilar data groups are transmitted to the base station, effectively reducing the transmission rate by eliminating redundant data vectors. Mohammadi et al. addressed the challenge of determining an optimal sampling rate in energy-harvesting WBANs to maintain self-sustainability without compromising service quality. They highlighted the unpredictability of harvestable energy rates and the variable nature of patient vital signs. Their paper proposed dynamic sampling-rate technique using deep reinforcement learning, where the Markov decision process framework captures both energy and data variability aspects [50]. Li et al. focused on minimizing energy consumption in WBANs during transmission sessions using amplify-and-forward (AF) relay through a hybrid supervised and DRL-based method that optimizes transmission parameters like power levels and block length, ensuring reliable, energy-efficient transmission under fluctuating channel conditions [51].

### 3.4. Coding-Based Approaches

Several coding-based schemes have been proposed to enhance the reliability of WBANs by adopting cooperative communication and network coding strategies. The first approach minimizes channel impairment and body fading effects, thereby reducing faults, bit error rates, and energy consumption. This method was demonstrated through a case study focused on remote sepsis monitoring, showcasing its effectiveness [12]. Another network coding approach was introduced to address load balancing in WBANs, dynamically adjusting packet redundancy levels via a Markov Decision Process (MDP) algorithm. This method significantly enhances data storage reliability and retrieval while optimizing energy consumption in the network [52]. GROWN, a data compression algorithm designed to reduce information redundancy in sensory data [53], operates in the following two stages: lossy data acquisition and lossless compression.

1. During the lossy phase, the data within an acceptable range are discarded, minimizing the bits required for compression in the subsequent lossless phase. The initial data point ($d_{i}$) is stored and compared with subsequent points. If the difference ($\Delta Z\;=\;|d_{i}-d_{j}|$) for a later data point ($d_{j}$) exceeds a predefined threshold ($t_{\tau }$), ΔZ is sent for compression and $d_{i}$ is updated to $d_{j}$; otherwise, $d_{j}$ is discarded by considering it a repetition.
2. In the lossless compression stage, GROWN employs a modified *exponential Golomb code*, a universal lossless encoding method suitable for coding non-negative values. Decoding tables are used at the server or base station for signal decompression and recovery of the original signal.

Another approach employs rateless channel coding to improve energy efficiency in Bluetooth networks for augmented reality [54]. Using a block-based rateless coding technique, this study demonstrated reduced energy consumption and enhanced video quality compared to traditional Bluetooth-based FEC schemes, especially under challenging channel conditions. This approach mitigates wireless channel errors and minimizes network redundancy, contributing to energy savings and improved video quality. Wang et al. focused on the use of network coding in IoMT systems and proposed a lightweight identity-based network coding scheme (IBNS) that enhances security while reducing computational overhead in signature verification, making it feasible for resource-constrained IoMT environments [55].

A logical XOR-based architecture [56] introduces a static approach for efficient wireless communication in WBANs while enhancing small-scale body area network reliability. Grounded in linear block coding theory, the architecture involves sensor nodes and relay nodes communicating with a destination. Each relay node strategically selects two sensors and three relay nodes to reduce the effective error rate in noisy environments. The XOR operation on data from these sources enhances data accuracy, with experiments demonstrating effectiveness, particularly in Rician channels. Finally, another coding-based approach, termed the Redundancy-Balanced Data Transmission Scheme (RDBT), emphasizes quality-of-service (QoS) parameters such as network lifetime, stability, throughput, and latency [57]. RDBT leverages compressive sampling on medical data characterized by low variation and high redundancy, mitigating energy loss from excessive data transmission. It categorizes sensor nodes based on data significance, enabling network balancing and responsive emergency measures. A selection algorithm for cooperative nodes in multi-hop communication considers residual energy, congestion control, and signal-to-noise ratio to optimize QoS. This approach demonstrates significant improvements in QoS, surpassing existing state-of-the-art methods.

## 4. Experimental Validation

We discuss the study design and implementation specifications, followed by a performance evaluation of the three baselines to optimize WBAN communication. The experiments are designed to demonstrate the effectiveness of data-driven approaches by incorporating machine learning, statistical methods, and coding-based techniques to address key challenges in WBANs, such as reliability (measured in terms of predictive accuracy of significant events) and quality of service (in terms of data loss and communication delay).

### 4.1. Study Design

#### 4.1.1. Simulation Platform

We developed a customized simulation platform using the Python discrete event simulation library called simulation in Python (SimPy) [58]. The simulator features an *event generator* (EG) module that associates physiological data collected using a prototype device (see Section 4.1.2) or sourced from the MIMIC clinical dataset (details discussed hereafter in Section 4.1.3), with location coordinates (sampled from a uniform random distribution) within a predefined simulation area representing the human body. Each event generated by the EG is a physiological data point and is tagged with a unique event identifier and location coordinates given by 〈ID,data,x,y〉. In our simulation setup, we deploy *n* sensors throughout the designated area, each with functionalities such as sensing, sending, and receiving event data in its vicinity, governed by a sensing range. These energy-constrained sensors can intercommunicate and transmit data to a local processing unit known as the base station (BS). Each biosensor node is implemented as a generator function within the SimPy simulation framework, simulating its real-world operations and energy consumption.

To evaluate three approaches for redundancy mitigation, the WBAN is simulated under various conditions in separate runs. These approaches are representative of statistical, machine learning or coding-based models designed to reduce data redundancy. The static sensors communicate through selective flooding, except when they are in the neighborhood of the base station (BS). In these cases, they directly forward the aggregated information received from their peers to the BS. For each baseline scenario, the BS assesses the following two key metrics: the predictive accuracy of anomalous clinical events and the energy savings achieved through the elimination or compression of redundant data. This dual evaluation provides a holistic understanding of each algorithm’s effectiveness in optimizing the predictive accuracy and energy efficiency of the WBAN.

#### 4.1.2. Real Data Collection Using a Wearable Biosensor Prototype Device

We developed a wearable prototype device to collect real data comprising blood oxygen level and pulse rate. It employs *photoplethysmography* (PPG), which is a non-invasive optical technique used to detect blood volume changes in the microvascular bed of tissue. It is used to measure various cardiovascular parameters, including heart rate and blood oxygen saturation (SpO_2_). Pulse oximetry leverages the differential light absorption properties of oxygenated hemoglobin (HbO_2_) and deoxygenated hemoglobin (Hb) at specific wavelengths. A fingertip probe transmits red (around 660 nm) and infrared (around 940 nm) light, chosen for their contrasting absorption rates by HbO_2_ and Hb. A photodetector measures the absorption at each wavelength as the light passes through the fingertip vasculature. The red-to-infrared light absorption ratio is used to estimate SpO_2_ through an empirically derived algorithm based on prior calibrations with blood samples of known oxygen saturation levels, providing continuous, non-invasive monitoring of SpO_2_. The oxygen saturation percentage is the ratio of $HbO_{2}$ in the arteries to the total *Hb*, i.e.,

$$SpO_{2}=\frac{HbO_{2}}{\mathrm{Hb}+HbO_{2}}\times 100\%$$

Oximetry devices utilize red and infrared LEDs in conjunction with photosensors to gauge the light intensity passing through fingertips. This intensity reduction is attributed to blood flow in veins, arteries, and tissues. Venous blood flow and tissue generate a stable DC signal, while arterial blood flow results in a less stable AC signal. The ratio of red-to-infrared light absorption producing AC and DC components is $R=\frac{\mathrm{AC}\mathrm{red}/\mathrm{DC}\mathrm{red}}{\mathrm{AC}\mathrm{ired}/\mathrm{DC}\mathrm{ired}}$, where *AC_red_*, *DC_red_*, *AC_ired_*, and *DC_ired_* are the AC and DC voltages from the absorption of red and infrared light, respectively. Lastly, given the voltage ratio (*R*) resulting from the absorption of red and infrared light (*AC_red_*), *SpO_2_* is $SpO_{2}=10-25R$.

**Figure 4:**
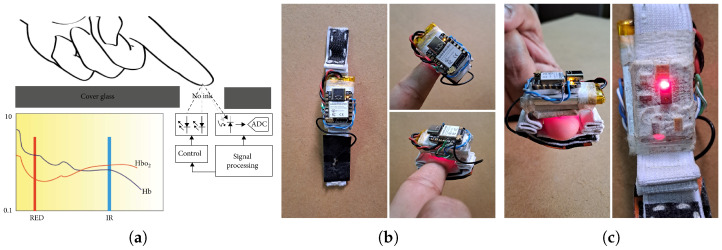
Prototype device for the collection of physiological data (namely, blood oxygen levels and pulse rates). (**a**) Block diagram and IC MAX30100 placement. (**b**,**c**) Circuit connection between the XIAO ESP32-S3 development board and the sensors.

The portable healthcare system utilizes an IC MAX30100 oximetry sensor to monitor health indicators. This integrated module features red and infrared LEDs, alongside signal conditioning components, all housed within a single package. By capturing voltage, the sensor module calculates *SpO_2_* levels and detects pulses. A schematic representation of the sensor module is shown in Figure 4a. The MAX30100 sensor module (manufactured by Maxim Integrated Products, Inc., a subsidiary of Analog Devices) positioned on thin body tissues like a fingertip, employs alternating red and infrared light from its second LED. This light is emitted onto the fingertip, where some is absorbed and reflected. The reflected light is detected by the photodiode on the IC MAX30100, generating a voltage that is transmitted to the integrated signal conditioner within the IC MAX30100. The resulting signal from the MAX30100 sensor module is conveyed to the XIAO ESP32-S3 using the I2C communication protocol. The system implemented for this study comprises the following open-source electronic components:

1. *ESP32-S3 microcontroller:* A cost-efficient tiny development board with dimensions of 21 mm × 17.5 mm, featuring Wi-Fi and Bluetooth capabilities and designed around the ESP32-S3 microcontroller. With 11 general-purpose input/output pins, support for interrupt/PWM/I2C/one-wire (except A0), and a single analog input (3.3 V max), it provides versatile functionality. The microcontroller is compatible with MicroPython, Arduino, and ESP-IDF, boasting a clock speed of up to 240 MHz, 8 MB PS-RAM, and 8 Mbytes of Flash storage. This device can be powered via a micro USB connection, accepting a 5 V input. Leveraging its Wi-Fi capabilities, the Seed Studio XIAO ESP32S3 facilitates the creation of applications for wireless data transfer to cloud infrastructure.
2. *MAX30100 pulse oximeter module:* With dimensions of 19 mm × 14.5 mm × 3 mm, this module is capable of embedding pulse oximetry and heart rate monitoring applications. Comprising two LEDs and a low-noise analog signal processing unit, it calculates heart rate and *SpO_2_* measurements using raw sensor data. Operating within a range of 1.8 V to 3.3 V, the sensor module includes an inbuilt voltage regulator. Equipped with a photo sensor, red LED (660 nm), and IR LED (880 nm) with radiation power of up to 9.8 mW and 6.5 mW, respectively, the sensor can achieve a high sample rate of up to 1000 Hz. Its programmable nature allows for precise control of a high data output. The module interfaces using the I2C protocol with a four-pin connection (VCC, GND, SDA, and SCL) to the MCU pins.
3. *PKCELL LIPO Battery*: A 350 mAh 3.7V lithium-ion polymer battery with dimension of 36 mm × 19.6 mm × 5.2 mm), a standard discharge rate of 0.2C, and a maximum continuous discharging current of 525 mA.
4. *Raspberry Pi 4 Model B*: This component features a Broadcom BCM2711, Quad-core Cortex-A72 (ARM v8) 64-bit SoC @ 1.8 GHz, 8 GB LPDDR4-3200 SDRAM, 2.4 GHz and 5.0 GHz 802.11ac [59] wireless, Bluetooth 5.0, gigabit Ethernet, operating on 5 V DC via a USB-C connector and consuming less than 500 mA.

This wearable sensor system targets the continuous monitoring of blood oxygen levels and pulse rates. The data are processed by the XIAO ESP32-S3 MCU from Seeed Studio, which features IoT communication technology. The XIAO ESP32-S3 MCU is a compact unit embedded with Wi-Fi and Bluetooth, along with integrated power management and battery protection features. A sensor attached to the MCU via I2C pins sends the collected data over short-range Wi-Fi to a gateway device hosted by a Raspberry Pi Single-Board Computer (SBC), where they are further processed. The IoT gateway receives data from the MCU every five seconds and transfers them to the cloud (see Figure 4b).

**Figure 5:**
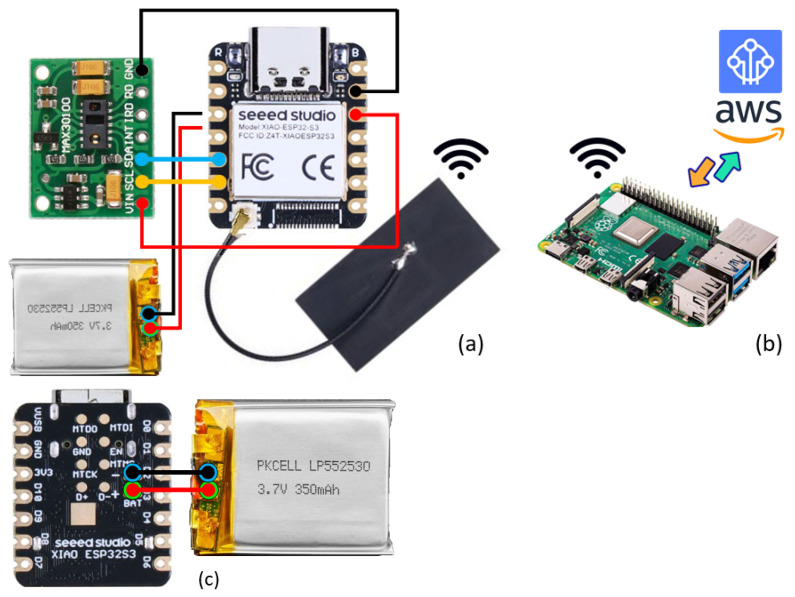
Components of the prototype. (**a**) Overview. (**b**,**c**) Gateway device connection with cloud and power supply to MCU.

Overall, the prototype is capable of various functions, including monitoring of patient data using sensors directly attached to the patient’s body. The sensors and MCU are integrated into a wearable device that patients can easily wear. This prototype can wirelessly transmit data to the cloud without the need for extra wires. An access point receives data from the wearable device via Wi-Fi. A schematic diagram of the circuit connection between the XIAO ESP32-S3 development board and the sensors is shown in Figure 5.

#### 4.1.3. MIMIC

The MIMIC-IV Database [14] is a repository of physiological health features and indicators obtained from intensive care patients. This repository contains various measurements of patients under critical care, namely electrocardiogram readings, blood oxygen level, respiratory rate, etc. The data are collected using medical equipment directly from the bedside of the patients. Patient identification related to the data is removed when placing the records in the database. We consider the following four physiological features for our analysis: heart rate (HR), pulse, breathing rate, and blood oxygen level (*SpO_2_*).

### 4.2. Implementation Specifications

#### 4.2.1. Data Labeling

We employed k-means clustering with k=2 to identify two clusters, where the majority class is classified as *not interesting* (0), while the minority class is marked as *interesting* (1). Furthermore, a generative algorithm called SMOTE [60] was used to augment the dataset size to 1000 data points, ensuring that 70% of the data points belong to the 0 class, while the remaining 30% are in the 1 class.

#### 4.2.2. WBAN Deployment

**Figure 6:**
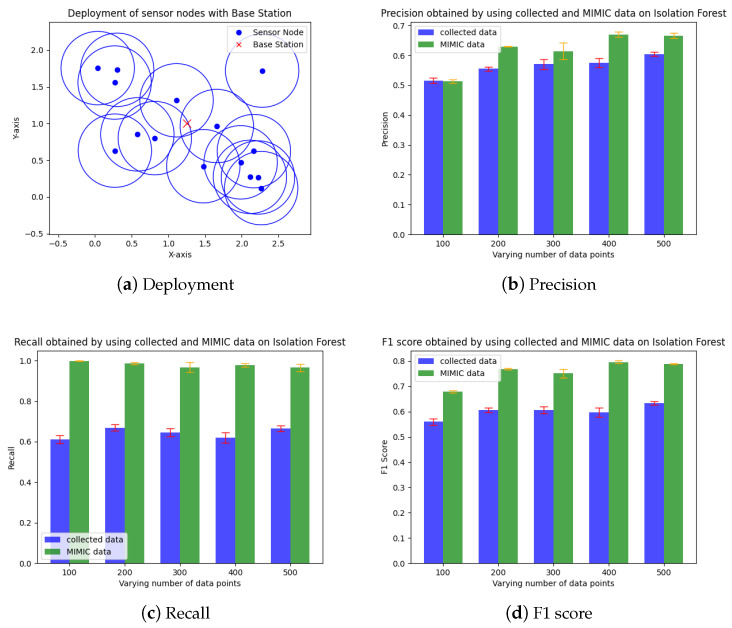
WBAN deployment and the predictive accuracy of the isolation forest-based redundancy mitigation approach [48] (MIMIC dataset).

The simulation setup is designed to ensure consistency in the evaluation of the three methods. Several physical parameters are kept constant for comparative analysis. Specifically, a total of 15 nodes are utilized, each with a sensing radius of 0.5 m. These nodes are randomly deployed within an area of approximately 2.5×2 square meters (see Figure 6a). The simulation duration is set to 200 min, providing a substantial period for data collection and analysis. For the statistical and machine learning-based approaches, a buffer size of 25 is implemented. This buffer size is chosen to balance between computational efficiency and accuracy. Additionally, the statistical method operates with a period of 10 min, allowing for periodic data aggregation and analysis. This setup ensures that the evaluation scores obtained from the three baseline approaches can be directly compared under identical conditions.

### 4.3. Comparative Analysis of Baseline Approaches

Finally, we evaluate the machine learning (ML) [48], statistical [49], and coding-based [53] approaches implemented using the customized simulator modeling a WBAN with biosensors exchanging biomedical datasets taken from the MIMIC dataset and real physiological data collected from six subjects through a hardware prototype. The procedures collected heart rate and blood oxygen data using non-invasive wearable sensing devices according to ethical standards and causing no risk to individuals. We discuss the performance of the stated approaches in terms of predictive accuracy and achieved compression averaged across 10 runs.

**Figure 7:**
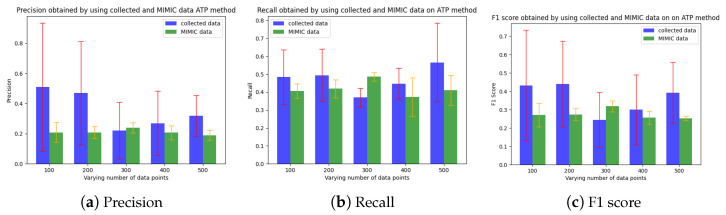
Predictive accuracy of the statistical redundancy mitigation approach [49] (collected data).

Figure 6 and Figure 7 show the precision, recall, and F1 score for the Isolation Forest (IF)-based ML approach and the ANOVA-based statistical counterpart (called ATP) on the MIMIC dataset, as well as the real collected data. The overall performance improves with the number of data points. The IF approach shows better predictive accuracy (F1 score∼0.80) with the MIMIC than with real data (F1 score∼0.55), whereas the ANOVA approach shows the opposite trend, (i.e., F1 score∼0.3 on MIMIC and F1 score∼0.45 on the collected data). Notably, the IF approach exhibits an improvement in precision for both datasets. We attribute its superior performance to its use of recursive partitioning, which effectively differentiates between redundant and non-redundant data. Furthermore, IF leverages an ensemble of trees during the training process, making it less susceptible to false positives. This ensemble method contributes to the high precision (see Figure 6b). Overall, the robustness of the IF-based approach to varying data distribution assumptions enhances its performance and reliability. Finally, we measure the *compression factor*, calculated as $1-\frac{\mathrm{number}\;\mathrm{of}\;\mathrm{compressed}\;\mathrm{bits}}{\mathrm{number}\;\mathrm{of}\;\mathrm{uncompressed}\;\mathrm{bits}}$, and the average number of bits transmitted by the coding-based approach (called GROWN) [53]. This approach takes advantage of the correlation between consecutive sensed values, which reduces the frequency of data to be transmitted during the lossy acquisition phase. The compression code also reduces the number of bits to be transmitted by the sensors. The GROWN approach is compared against the average number of bits transmitted without compression, which is based on a 14-bit ADC with higher resolution.

**Figure 8:**
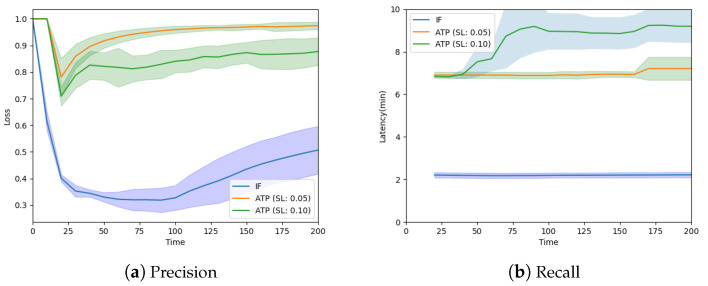
Predictive accuracy of the statistical redundancy mitigation approach [49].

The analysis (on 500 data points) of the QoS of the IF and ANOVA approaches shows similar trends as the earlier analysis on predictive accuracy. The *data loss rate* is measured in terms of the proportion of unique events not received by the BS, while *latency* is measured in terms of the time elapsed between the occurrence of the event and its receipt at the BS. For ANOVA, we analyze the effects of two significance levels (SLs), i.e., 0.05 and 0.10, on the overall performance. Figure 8 shows the loss for ANOVA and latency to be significantly higher than for IF. Since a higher SL makes it easier to reject the null hypothesis that the two points are similar, ANOVA with an SL of 0.10 exhibits marginally improved results relative to those achieved with an SL of 0.05.

**Figure 9:**
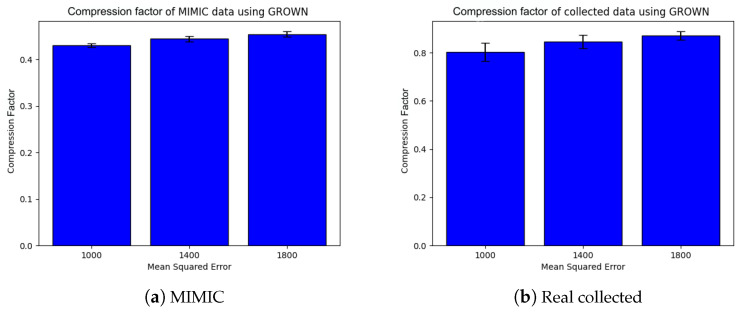
Redundancy minimization through compression achieved using GROWN [53].

**Figure 10:**
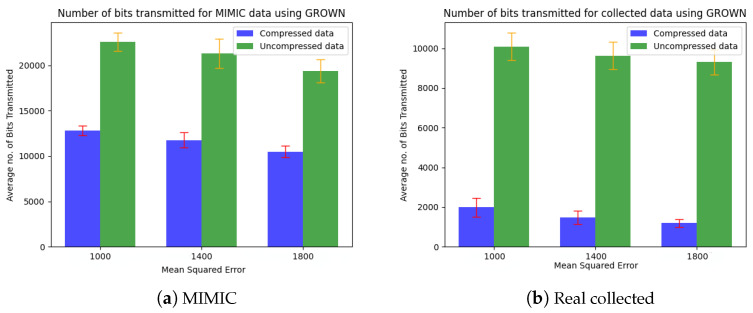
Reduction in the number of transmitted bits achieved using GROWN [53].

Two consecutive data points sensed by a given node are considered the same if the mean squared error (MSE) between them is within a predefined threshold range. As illustrated in Figure 9 and Figure 10, an increase in the MSE threshold results in greater compression, captured in terms of an increased compression factor and a reduction in the number of transmitted bits for both MIMIC and the real collected data.

## 5. Discussions

Future WBANs, especially those incorporating lightweight, energy-constrained biosensor nodes, must carefully balance reliability with energy and storage efficiency. As highlighted in this survey, energy efficiency is paramount due to the limited battery life of biosensor nodes [61]. Leveraging the inherent spatial and temporal patterns in biomedical data streams can enhance both data transmission reliability and robustness against failures while managing energy consumption. To address these challenges, strategies like *adaptive correlation-based optimization* can dynamically adjust communication parameters based on network conditions and energy levels. Additionally, energy-efficient encoding, decoding, and data aggregation methods will be critical in minimizing storage overhead. Optimal node placement, utilizing overlapping radio ranges to provide redundancy, can improve network reliability and ensure continuity in cases of node failure [62]. In WBANs, effectively managing spatial and temporal correlations minimizes data loss risks and ensures uninterrupted monitoring, which is especially vital in critical healthcare settings.

The emergence of *software-defined wireless body area networks* (SD-WBANs) represents another significant advancement in the field, offering a flexible and programmable network architecture where a centralized controller can dynamically regulate network topology and traffic flow [63]. This is particularly useful in scenarios where the WBAN exhibits heterogeneity in terms of power sources and configuration [64]. Understanding spatial and temporal trends in data streams becomes particularly important in SD-WBANs, as they can substantially enhance fault tolerance in time-critical applications. By introducing multiple controllers within the SD-WBAN framework, the network can achieve higher levels of reliability and resilience [61]. These additional controllers can act as backup nodes, taking over control and data transmission responsibilities in the event of a failure, thereby ensuring uninterrupted service and reducing the risk of data loss. The centralized nature of SD-WBANs allows for sophisticated management strategies, such as real-time monitoring and adaptive reconfiguration based on data correlations, optimizing resource utilization, and maintaining high-quality service levels. Integrating machine learning algorithms to preemptively address potential failures can also enhance their effectiveness in SD-WBANs.

Another security consideration is the possibility of compromising nodes to push incorrect health information. In future WBAN applications, it will be crucial to adopt consensus or data fusion techniques to minimize adverse health outcomes from misreporting. Compromised nodes can potentially provide false data that may lead to incorrect diagnoses or treatments, posing significant risks to patient safety. Consensus, as well as data fusion techniques, involve multiple nodes reaching an agreement on the reported data, ensuring that a single compromised node cannot influence the overall system [27]. These techniques can help validate the accuracy of the data by comparing inputs from multiple biosensors and identifying possible anomalies or inconsistencies. By requiring a majority agreement before data are considered valid, consensus mechanisms can effectively mitigate the impact of compromised nodes. Incorporating these security measures into the design of future WBANs will be essential for maintaining the integrity and reliability of health monitoring systems. By leveraging spatial and temporal trends to improve network performance and enhance security, researchers can develop robust WBANs capable of providing accurate and reliable health data, even in the presence of potential threats.

## 6. Conclusions

This survey examined strategies to address inherent spatial and temporal trends in biomedical data, focusing on time-critical optimization goals such as robustness, optimized placement, routing approaches, sampling rate calibration, machine and statistical learning, and coding-based methodologies in wireless body area networks (WBANs). Our analysis, utilizing a combination of the MIMIC clinical database and physiological data collected using a prototype biosensing device built by our team, reveals that machine learning (ML) approaches, particularly the isolation forest algorithm, significantly outperform statistical methods in leveraging these correlations. Our results indicate that larger sample sizes substantially enhance the predictive accuracy in identifying anomalous readings in healthcare data. These insights highlight the importance of advanced ML techniques and adequately sized datasets in improving energy efficiency, security, reliability, and robustness in WBANs. We conclude with a discussion on the future of WBAN research to enhance the quality of service and accuracy for real-world healthcare solutions.
